# The use of rotational thromboelastometry parameters in understanding the coagulopathy following hump-nosed viper (*Hypnale spp*) bites: a preliminary study

**DOI:** 10.1186/s40794-022-00186-2

**Published:** 2023-01-02

**Authors:** Bhawani Yasassri Alvitigala, Lallindra Viranjan Gooneratne, Iresha Dharmasena, Nuwan Premawardana, Manujasri Wimalachandra, Miyuru Weerarathna, Roopen Arya, Ariaranee Gnanathasan

**Affiliations:** 1 Department of Pathology, Faculty of Medicine, University of Colombo, Colombo 08, Sri Lanka; 2 Department of Hematology, Teaching Hospital Anuradhapura, Anuradhapura, Sri Lanka; 3 Department of Clinical Medicine, University of Rajarata, Anuradhapura, Sri Lanka; 4 Department of Hematological Medicine, King’s College Hospital, Denmark Hill, SE5 9RS London, UK; 5 Department of Clinical Medicine, Faculty of Medicine, University of Colombo, Colombo 08, Sri Lanka

**Keywords:** Coagulopathy, Hump-nosed viper, Rotational thromboelastometry, WBCT20

## Abstract

**Background:**

Hump-nosed vipers (HNV; *Hypnale spp*) are one of the medically important venomous snakes in Sri Lanka and South-Western regions of India. The haemostatic dysfunction due to HNV bites is poorly characterized by standard diagnostic tests performed to identify coagulopathy. We aimed to determine the usefulness of rotational thromboelastometry (ROTEM) parameters compared to 20-minute whole blood clotting test (WBCT20) and prothrombin time (PT) in understanding the coagulopathy of HNV bites.

**Methods:**

Twenty-three HNV snakebite patients in a prospective study of 127 consecutive snakebites were recruited. After recording details of the clinical presentation, PT/international normalized ratio (INR), WBCT20 and ROTEM *delta* were performed at presentation.

**Results:**

In this preliminary study, none of the patients had clinically apparent bleeding. Coagulopathy was detected by either WBCT20, INR or ROTEM in 13 HNV patients. Eleven had a coagulopathy detectable by ROTEM (either abnormal EXTEM-CT, INTEM-CT or FIBTEM-MCF) but with negative WBCT20. Of them, only two had prolonged INR values. Two patients had positive WBCT20 but with normal ROTEM and INR values. The remaining 10 patients did not show any coagulopathy either by INR, ROTEM or WBCT20.

**Conclusion:**

In this preliminary study with small number of sample size, ROTEM parameters appeared to be more sensitive to subtle changes in coagulation compared to WBCT20. The clinical utility of detecting these changes and their usefulness in managing snakebite should be explored further in a larger study.

## Introduction

According to the current updates, there are 108 species of snakes in Sri Lanka of which 51 are endemic [1]. Among the venomous and medically important snakes in Sri Lanka, the hump-nosed viper (HNV; *Hypnale spp*) is considered the most common accounting for 22–77% as reported in different studies [2–4]. The HNV, or Merrem’s hump-nosed pit viper, is considered a medically important snake due to its serious, often unpredictable, local and systemic effects of envenoming [2–4]. Three species of genus: *Hypnale* have been described: *H hypnale, H zara* and *H nepa* of which the latter two are only found in Sri Lanka while the former is also found in the Western Ghats of South India [5]. The venom composition and toxic effects of the three species have been shown to be similar [6, 7].

Varying incidences (2.6-39%) of haematotoxicity have been reported in HNV bites [2, 8]. The coagulopathy can vary from asymptomatic derangement of clotting tests to life threatening bleeds [6]. The mechanism of coagulopathy in HNV is incompletely understood. Activation of clotting factors in the common pathway resulting in depletion of clotting factors due to consumption can result in an increased bleeding risk [6, 8]. Thrombin-like enzymes found in HNV venom can lead to the cleavage of fibrinogen into fibrinopeptides without the production of fibrin monomers which are essential for a stable clot. In addition, thrombotic microangiopathy has also been described in HNV bites [9].

The 20-minute Whole blood clotting test (WBCT20) is an inexpensive bedside test that can indicate coagulopathy in snakebite envenoming [9]. However, as would be expected from a crude bedside test, it does not provide any information on the type of coagulopathy present in the patient [6]. In most cases, HNV bite-induced coagulopathy could not be diagnosed by WBCT20 or prothrombin time (PT) due to their several associated limitations [7]. Despite this, WBCT20 is routinely used to detect envenoming in patients following HNV bites. Rotational thromboelastometry (ROTEM) is a novel and sensitive test that monitors the dynamic and physical characteristics at different phases of clot formation and lysis using a graph from the beginning of coagulation to the point of fibrinolysis [10]. Coagulation cascade can be monitored by three ROTEM tests: intrinsic pathway by IN-TEM, extrinsic pathway by EX-TEM and fibrinolytic pathway by FIB-TEM [11]. ROTEM has been shown to be better at detecting coagulopathy and varying degrees of haemotoxicity, including venom induced consumption coagulopathy (VICC), compared to PT and WBCT20 [12].

The current report is part of a larger study which was designed to compare ROTEM parameters with the WBCT20 in venomous snakebites to better understand its utility in snakebite management.

## Methodology

**Fig. 1:**
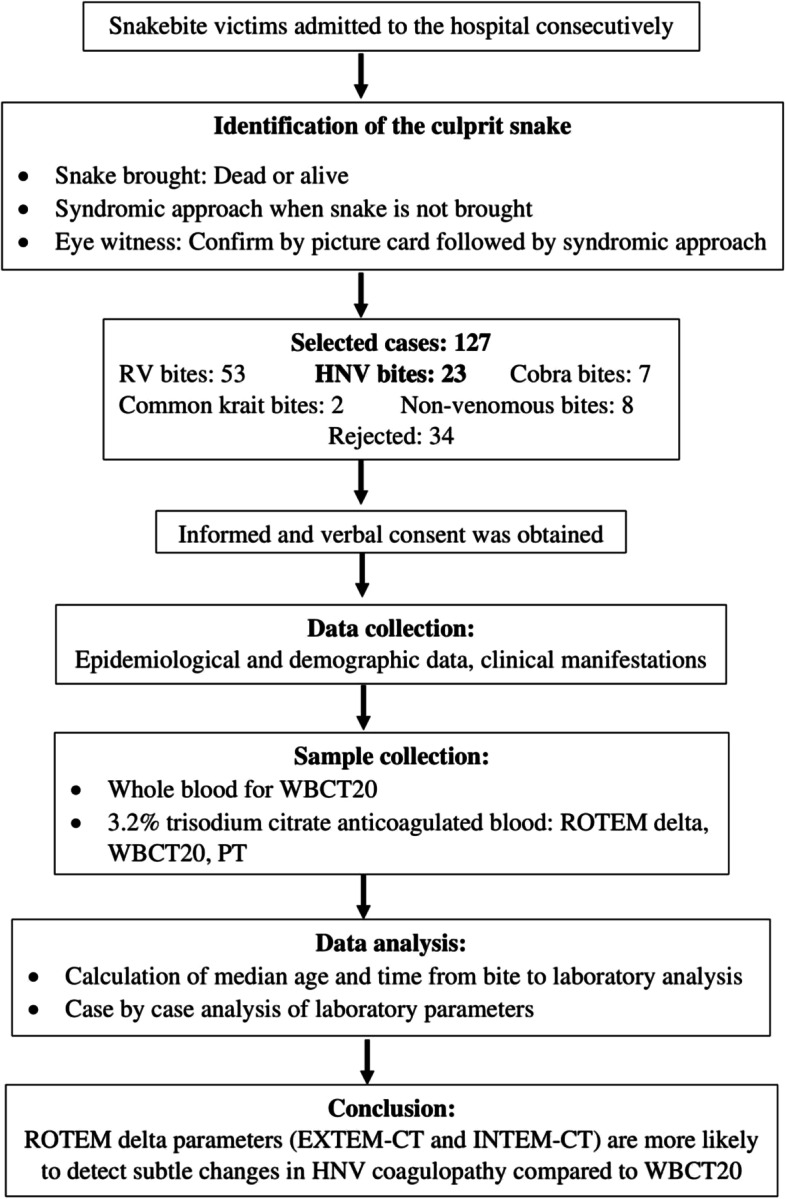
Summary of the present study (CT, clotting time; EXTEM, extrinsic pathway thromboelastometry; HNV, hump-nosed viper; INTEM, intrinsic pathway thromboelastometry; PT, prothrombin time; ROTEM, rotational thromboelastometry; RV, Russell’s viper; WBCT20, 20 min whole blood clotting test)

A prospective study was carried from 2016 to2017, at the Anuradhapura Teaching Hospital, a key hospital for snakebite management in the North Central Province of Sri Lanka. Of 127 snakebites the responsible snake was not clearly identified or complete data sets were not available in 34. There were 53 Russell’s viper bites, 23 HNV bites, 7 cobra bites, 2 common krait bites and 8 non- venomous snakebites identified. This article describes the patients with HNV bites (summarized in Fig. 1).

Ethical approval for the study was granted by the Ethics Review Committee, Faculty of Medicine, University of Colombo (EC-12-30).

### Identification of the culprit snake

The culprit snake was identified accurately if the snake was brought to the hospital (alive or dead) based on the distinctive features of HNV [13]. When the snake was not brought, identification was based on the syndromic approach. A method which has shown a specificity > 95% in identifying snake species in the local setting [14]. Once the clinical syndromes of presentation were matched to the identified snake based on the algorithm, such cases were enrolled in the study. When an eyewitness was present, snake identification using the syndromic approach was complemented by matching the observed picture cards of locally prevalent snakes [14]. Application of the syndromic approach and identification of live/ dead snakes was performed by one of the investigators, a consultant physician, trained in this process. HNV species identification was not performed. Hence, all the identified cases were defined up to genus level.

### Data collection

Demographic characteristics, epidemiological data, clinical manifestations, and time from bite to laboratory testing were recorded. Some of this information was collected to aid the identification process of the culprit snake. Clinical manifestations at presentation were recorded under five categories. They were, haematological (bleeding), neurological (blurred vision, ptosis, ophthalmoplegia, drowsiness, paresthesia, myotoxicity, dizziness), nephrological (renal impairment, acute kidney injury (AKI), local effects (swelling, pain at the bite site, tenderness, lymphadenopathy, blistering, necrosis) and non-specific features (nausea, vomiting, abdominal pain, anxiety and presyncope). AKI was evaluated by urinalysis, blood film analysis and renal function tests (RFTs) and myotoxicity by clinical evaluation of muscle pain, creatine phosphokinase levels, full blood count and RFTs.

### Obtaining patient consent

Informed and verbal consent was obtained from the patient prior to enrollment, while written consent was obtained prior to obtaining blood samples.

### Collection of blood samples

Three blood samples were collected from a single venipuncture to 3.2% tri-sodium citrate tubes and analyzed on a ROTEM *delta* (Instrumentation Laboratory, Munich, Germany) at the haematology laboratory of the hospital soon after collection. Blood samples for WBCT20 and PT/ international normalized ratio (INR) were collected at the time of admission as routine tests for snakebite patients and an additional sample for ROTEM delta was collected after obtaining written consent on the same day (within 24 h). PT and ROTEM *delta* were performed at the haematology laboratory of the hospital soon after collection. The instrument was validated for routine clinical use by the laboratory of the hospital.

### Sample analysis

WBCT20 analysis was performed adhering to the standardized procedure by trained personnel. 1 mL of whole blood was collected into a clean, dry, 5 mL borosilicate glass tube and kept undisturbed for 20 min, after which the tube was inverted to determine if the blood had clotted [15].

PT was performed on an ACL elite-Pro coagulation analyzer used for clinical services in the hospital with daily internal quality checks and regular participation in an external quality assurance programme.

Three assays were performed by ROTEM *delta.* Clotting time (CT) of INTEM and EXTEM and maximum clot firmness (MCF) of FIBTEM were assessed with a run time of 70 min.

The decision to administer antivenom was made by the medical team attending to the patient, by using a combination of clinical and laboratory features based on local,national and regional guidelines on snakebite management [16]. This decision was not influenced by results of PT/INR and ROTEM tests done for the study.

Established reference ranges were used for CT and MCF of INTEM, EXTEM and FIBTEM parameters. INR > 1.4 was considered as abnormal. The median was statistically analyzed using SPSS 23.0.0.0 at 95% confidence interval (CI) (released 2015, IBM statistics for Windows version 23, IBM Corp., Armonk, NY).

## Results

The majority of the HNV bite victims were males (87%) and the median age of the victims was 50 years (range: 13–70 years). Bites occurred from dusk to dawn. (range: 1800 h to 0600 h) with the location of bite distributed evenly between farmlands and immediate home environments (home gardens). Median time from bite to laboratory testing was 12 h ( IQR: 18 h).

The culprit snake was brought to hospital in 8 (35%) HNV bites while 6 (26%) bites were identified using the syndromic approach by the treating physician and were further confirmed based on the identification of the snake by picture cards. The balance cases (9 cases; 39%) were identified using the syndromic approach alone.

No bleeding manifestations were observed in any of the victims. Four patients had AKI, non-specific effects and local effects were seen in all victims (Table 1).

**Table 1:** HNV case scenarios

**Age **(years)**/ Gender**	**INR **<1.4	**INTEM-CT **(sec)	**EXTEM-CT **(sec)	**FIBTEM-MCF **(mm)	**Platelet count **(×10^9^/ L)	**WBCT20**	**AVS**	**Summary**	**Clinical syndrome**
70/F	1.04	119	58	15	275	Clotted	Not given	Clotted WBCT20 and normal ROTEM	AKI, NS+, LE+
65/ M	0.97	171	64	12	135				AKI, NS+, LE+
58/ M	1.13	225	61	16	226				NS+, LE+
33/ M	1.01	155	69	16	-				NS+, LE+
13/ M	1.14	170	61	10	-				NS+, LE+
63/ M	0.906	187	55	10	183				NS+, LE+
34/ M	1.03	143	50	12	322				NS+, LE+
59/ M	1.15	170	77	13	-				NS+, LE+
57/ F	1.07	129	56	19	-				NS+, LE+
37/ F	0.99	185	79	14	263				NS+, LE+
56/ M	4.88 ↑	165	97 ↑	18	221	Clotted	Not given	Clotted WBCT20 and abnormal ROTEM.	NS+, LE+
50/ M	1.16	177	90 ↑	11	239				NS+, LE+
40/ M	1.06	130	102 ↑	10	223				NS+, LE+
40/ M	1.00	178	83 ↑	24	-				NS+, LE+
48/ M	1.4	151	83 ↑	12	288				NS+, LE+
50/ M	0.91	257 ↑	64	10	194				AKI, NS+, LE+
57/ M	0.963	2510 ↑	66	13	150				NS+, LE+
31/ M	1.14	389 ↑	77	10	264				NS+, LE+
15/ M	1.17	347 ↑	73	17	267				NS+, LE+
52/ M	1.00	263 ↑	54	17	19 ↓		Given		AKI, NS+, LE+
40/M	1.79 ↑	312 ↑	2285 ↑	3 ↓	122 ↓				NS+, LE+
29/ M	1.06	109	65	9	259	Non-clotted	Not given	Non-clotted WBCT20 and normal ROTEM	NS+, LE+
59/M	1.03	240	70	7	130				NS+, LE+

*CT *Clotting time, *EXTEM* Extrinsic pathway thromboelastometry, *FIBTEM* Fibrinolytic pathway thromboelastometry, *INR* International normalized ratio, *INTEM* Intrinsic pathway thromboelastometry, *MCF* Maximum clot firmness, *ROTEM* Rotational thromboelastometry, *WBCT20* Whole blood clotting test

↑Abnormally high, ↓Abnormally low, *F*= female, *LE* Local effects, *M* Male, *NS* Non-specific effects

(Reference ranges: Platelet count 150-450 ×10^9^/ L; INTEM-CT 100-240 sec; EXTEM-CT 38-79 sec; FIBTEM-MCF 9-25 mm)

The patients were categorized into groups based on the WBCT20 and ROTEM abnormalities (Table 1). Although none of the patients had clinically apparent bleeding, coagulopathy was detected by either WBCT20, INR or ROTEM in 13 HNV patients. Eleven had a coagulopathy detectable by ROTEM (either abnormal EXTEM-CT, INTEM-CT or FIBTEM-MCF) but with clotted WBCT20. Of them, only two had prolonged INR values. Two patients had non-clotted WBCT20 but with normal ROTEM and INR values. The remaining 10 patients did not show any coagulopathy either by INR, ROTEM or WBCT20.

## Discussion

This cohort’s epidemiological features were the same as those previously reported [1, 17–19]. The presence of more male victims is a reflection of the dominant workforce in farming communities. The majority of the patients were able to see a tertiary-level hospital facility that specializes in treating snakebites relatively quickly (median 12 h; IQR: 18 h) after being bitten, which was both interesting and reassuring. A study done in the Kurunegala district of Sri Lanka revealed a comparable, early health-seeking behavior, reporting a median time of 45 min (IQR 30–90 min) for a snake bite victim to arrive at a primary care hospital [20]. Additionally, they noted that 3.7% patients received indigenous care before visiting the hospital. Our study lacked data on, transfers from primary or secondary care facilities or indigenous treatments before patients presented to the hospital.

The above results indicate that ROTEM *delta* is more sensitive than WBCT20 and INR to identify coagulopathy. The single case of non-clotted WBCT20, despite normal ROTEM and INR without bleeding is unexpected. It may have been due to a deviation from the standard protocol as the WBCT20 is prone to false positive results even due to small preanalytical errors [9, 21]. The WBCT20 is still widely used as an indicator of envenoming, despite its reported low sensitivity of 40% [21, 22]. Its sensitivity can be improved to 80% when the test is performed under standardized conditions [23]. The need for an alternative bedside test which addresses its shortcomings has been highlighted [24]. We have previously demonstrated that EXTEM-CT in ROTEM has a 93% sensitivity and 92% accuracy in detecting Russell’s viper envenoming over WBCT20 [15].

The antivenom available in Sri Lanka is sourced from India and is effective in envenomation by Indian species of *Naja naja*, *Bungarus caeruleus*, *Daboia russelii* and *Echis carinatus*. The lack of efficacy of this polyvalent antivenom in HNV has been shown in many studies [6, 17, 24]. Despite this, many physicians use this antivenom in HNV bites [4]. This practice can be justified, as differentiating between the juvenile Russell’s viper and HNV can be challenging at times. Two patients in the present study were also treated with antivenom, although, one of them had normal WBCT20, INR and ROTEM results. The other had abnormal INR (1.79), EXTEM-CT (2285 s) and FIBTEM-MCF (3 mm) at presentation, prior to antivenom administration. Interestingly, the latter showed an improvement in the INR (0.67), EXTEM-CT (127 s) and FIBTEM-MCF (5 mm) within 24 h of receiving antivenom. Since the Indian Polyvalent antivenom has no benefit against *Hypnale spp* envenoming, the improvement may have been due to misidentification of the snake. On both occasions the culprit snake was not brought and was identified using the syndromic approach only.

Systemic effects of HNV bites are rare; coagulopathy has been described as the second commonest [8]. However, different studies have shown varying incidences of this complication. In a descriptive observational study involving 1543 HNV bite patients, only 59 (3.8%) were found to have coagulopathy by WBCT20, activated partial thromboplastin time (APTT) and PT/INR [4]. In another study, PT, APTT, Von Willebrand factor levels, clotting factor levels and D-dimers demonstrated a mild coagulopathy in all 80 patients evaluated, while WBCT20 was positive in only one patient [25]. It has also been reported that plasma containing *Hypnale spp.* venom cause weak, unstable and transient fibrin clots in thromboelastography [26]. Different frequencies of abnormal WBCT20 results were found in the three different sub species of HNV in Sri Lanka [27, 28]. This is also a possible contributor to the observed variation in coagulopathy among studies as the 3 species vary in their geographical distribution within the island [25, 29]. The results of these studies demonstrate that, mild HNV coagulopathy cannot be detected by WBCT20 possibly due to poor sensitivity and lack of standardization of the test. Furthermore, in patients presenting with clinically significant bleeding, understanding the exact coagulation derangement will be important in the management [8].

**Fig. 2:**
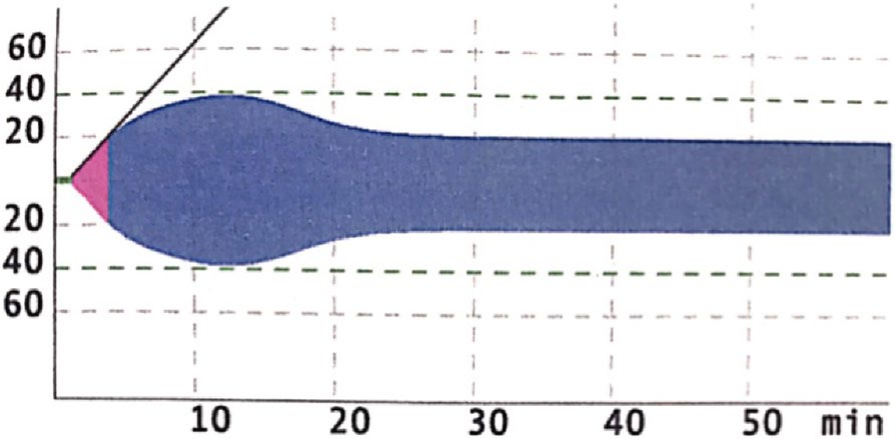
EXTEM of a 48-year-old male post HNV bite showing features of hyperfibrinolysis. ML = 45% (EXTEM, extrinsic pathway thromboelastometry; HNV, hump-nosed viper; ML, maximum lysis)

In contrast, when there is no clinically apparent bleeding despite abnormal coagulation, the need for a highly sensitive test to detect coagulopathy in HNV patients is obscure. ROTEM will be useful to understand the mechanism of bleeding in patients with VICC. In our study, this was seen in one patient who had hyperfibrinolysis on ROTEM, however, without apparent bleeding. (Fig. 2) Routine use of ROTEM in rural health care facilities in Sri Lanka is challenging due to high cost of the instrument (US$25,276) and high cost per test (approximately US$13) when compared to PT (US$2) and WBCT20 which only requires a borosilicate glass tube. However, this could be counterbalanced by applying ROTEM in other clinical settings such as obstetrical hemorrhage, liver transplantation, trauma and surgical bleeding [10].

This study has several limitations. Firstly, in a majority of cases the snake was not available for the medical team to reliably identify the species. Species identification was done mainly using the syndromic approach and identifying the snake that was seen at the time of bite on a chart of photographs. It would have been ideal if the type and concentration of the specific venom could have been detected to identify the snake accurately. Secondly, although the time from bite to laboratory testing was available, information on any treatment such as “traditional or native” decoctions used prior to presenting to hospital was unavailable. Further, there were no records on patient transfers from primary care hospitals. Thirdly, this was a preliminary study with a small sample size which was not adequate to demonstrate a statistical significance.

One of the major limitations of this preliminary study was that the fibrinogen concentrations of the patients were not assessed. In VICC caused by *Hypnale* spp. venom, excessive fibrinogenolysis is a key feature which could be indicated by increased levels of fibrinogen degradation products and decreased levels of fibrinogen [25, 29]. However, the MCF of FIBTEM which is a measure of the fibrinogen generation of the clot was available in all patients. This parameter was normal in all 22 patients except the patient who had grossly abnormal coagulation parameters without clinical bleeding.

In this preliminary study including small sampling of patients with HNV bites, we have demonstrated that ROTEM parameters were more likely to pick up subtle changes in coagulation when compared to conventional tests of coagulation. The clinical utility of detecting these changes and their usefulness in managing snakebite should be further explored. It would be prudent to validate these preliminary findings in a prospective study that will address the shortcomings described above, and thereby establish a more useful and accurate bedside test to determine HNV coagulopathy.

## Data Availability

Relevant data will be made available on request.

## Abbreviations

AKI: Acute kidney injury
CT: Clotting time
EXTEM: Extrinsic pathway thromboelastometry
FIBTEM: Fibrinolytic pathway thromboelastometry
HNV: Hump-nosed viper
INR: International normalized ratio
INTEM: Intrinsic pathway thromboelastometry
MCF: Maximum clot firmness
PT: Prothrombin time
RFT: Renal function test
ROTEM: Rotational thromboelastometry
VICC: Venom induced consumptive coagulopathy
WBCT20: Whole blood clotting test
